# Small Sample Sizes Yield Biased Allometric Equations in Temperate Forests

**DOI:** 10.1038/srep17153

**Published:** 2015-11-24

**Authors:** L. Duncanson, O. Rourke, R. Dubayah

**Affiliations:** 1 Department of Geographical Sciences, University of Maryland, College Park, 2181 Lefrak Hall, University of Maryland, College Park, MD, 20742; 2 Biospheric Sciences Lab, Code 618, NASA Goddard Space Flight Center, Greenbelt, MD 20771; 3 AMSC, Department of Mathematics, University of Maryland, College Park, 0209 Mathematics Building, University of Maryland, College Park, MD, 20742

## Abstract

Accurate quantification of forest carbon stocks is required for constraining the global carbon cycle and its impacts on climate. The accuracies of forest biomass maps are inherently dependent on the accuracy of the field biomass estimates used to calibrate models, which are generated with allometric equations. Here, we provide a quantitative assessment of the sensitivity of allometric parameters to sample size in temperate forests, focusing on the allometric relationship between tree height and crown radius. We use LiDAR remote sensing to isolate between 10,000 to more than 1,000,000 tree height and crown radius measurements per site in six U.S. forests. We find that fitted allometric parameters are highly sensitive to sample size, producing systematic overestimates of height. We extend our analysis to biomass through the application of empirical relationships from the literature, and show that given the small sample sizes used in common allometric equations for biomass, the average site-level biomass bias is ~+70% with a standard deviation of 71%, ranging from −4% to +193%. These findings underscore the importance of increasing the sample sizes used for allometric equation generation.

Global forests cover approximately 30% of the land’s surface and have been estimated to store approximately 1.03 million megatons (Mt) of carbon^1^. Estimates of forest carbon content are not only important inputs to global carbon cycle and climate models, but integral to the mitigation of climate change through market-based initiatives such as Reduced Emissions from Deforestation and Degradation (REDD + )^2^,^3^. Much research in the field of forest carbon mapping has focused on the development of remote sensing approaches to map biomass; in particular examining statistical methods for bringing together field and satellite data that permit estimation of carbon stocks and their associated errors^4^,^5^,^6^,^7^,^8^,^9^,^10^,^11^. Considerably less attention has been given to the accuracies of the field-based estimates themselves^12^,^13^,^14^.

Virtually all field estimates of biomass rely on the application of allometric equations relating properties that can be measured in the field, such as stem diameter and height, to individual tree carbon stock^15^. These allometric equations are typically derived through the destructive sampling of a relatively small number of trees that have been measured and felled to assess their carbon stock. Equations are generated either for individual species^16^,^17^, groups of species^15^,^18^ or for geographic regions^19^,^20^. In the tropics, it has been demonstrated that allometric equation selection is the primary source of error in tropical field-based biomass estimates, and that the sample size of trees used to generate allometric equations was one of the primary drivers of this error^12^,^13^,^14^.

In temperate systems, allometric equations generated with small sample sizes are widely applied, with popular allometric equations being built with average sample sizes of 23^15^, 81^21^,^22^ or a few hundred^23^,^24^,^25^,^26^ destructively sampled trees per species. The effects of these relatively small sample sizes on allometric equation development in temperate systems are unknown because data to test allometric parameter sensitivity to sample size have been previously unavailable. However, new remote sensing methods allow the extraction of individual tree properties across wide areas, with large sample sizes, and thus provide an alternate means to assess the impact of sample size on allometric parameterization^27^. LiDAR remote sensing is now a well-established means of obtaining individual tree height and crown dimensions, not just for a few hundred trees, but for millions of trees across entire landscapes. This is particularly feasible in temperate forests with relatively simplistic structures and open canopies where LiDAR has been demonstrated to most accurately extract individual crown information^27^.

Allometric equations can be developed between any number of tree structural properties, not just related to biomass but also describing relationships such as between stem diameter and height or crown diameter and height^28^,^29^,^30^. In this study we focus on an analysis of the impact of sample size on the generation of allometric relationships between crown radius and tree height, because we do not have direct measurements of biomass with LiDAR. However, these results are important for biomass estimation in two ways. First, if crown radius is an appropriate proxy for stem diameter, which we assert, then these results have direct implications for biomass estimates that rely on diameter to height relationships^14^,^19^,^20^. Second, these results have implications for biomass estimates that are not dependent on height, but estimated from diameter alone, because tree structural allometric relationships have been demonstrated to scale in the form of power laws^31^,^32^,^33^, and thus we can translate between structural allometries to infer how the error on fitted parameters for any one allometric equation will potentially impact another. We fit parameters to allometric equations relating LiDAR-derived height and crown radius, and translate observed biases in fitted parameters to expected biases between stem diameter and biomass through the application of regionally applicable empirical scaling relationships.

## Results

### The Effects of Sampling on Allometric Parameters

**Figure 1:**
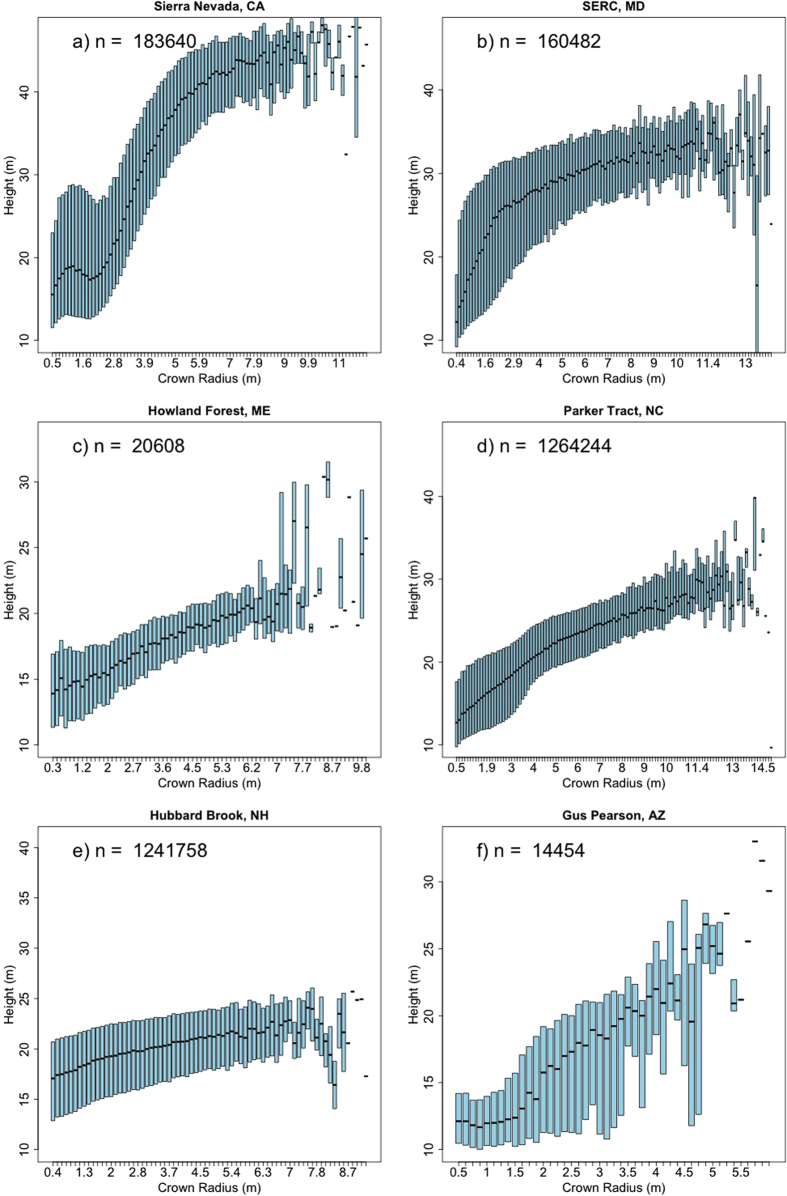
The relationships between tree height and crown radius at the six study sites. The number of delineated crowns at each site is displayed in the top left of each figure. The blue bars represent the 10^th^ to 90^th^ percentiles of heights in each crown radius bin, while the black lines represent the median tree height in each bin, at (**a**) Teakettle, (**b**) SERC, (**c**) Howland, (**d**) Parker Tract, (**e**) Hubbard Brook and (**f**) Gus Pearson, respectively.

**Figure 2:**
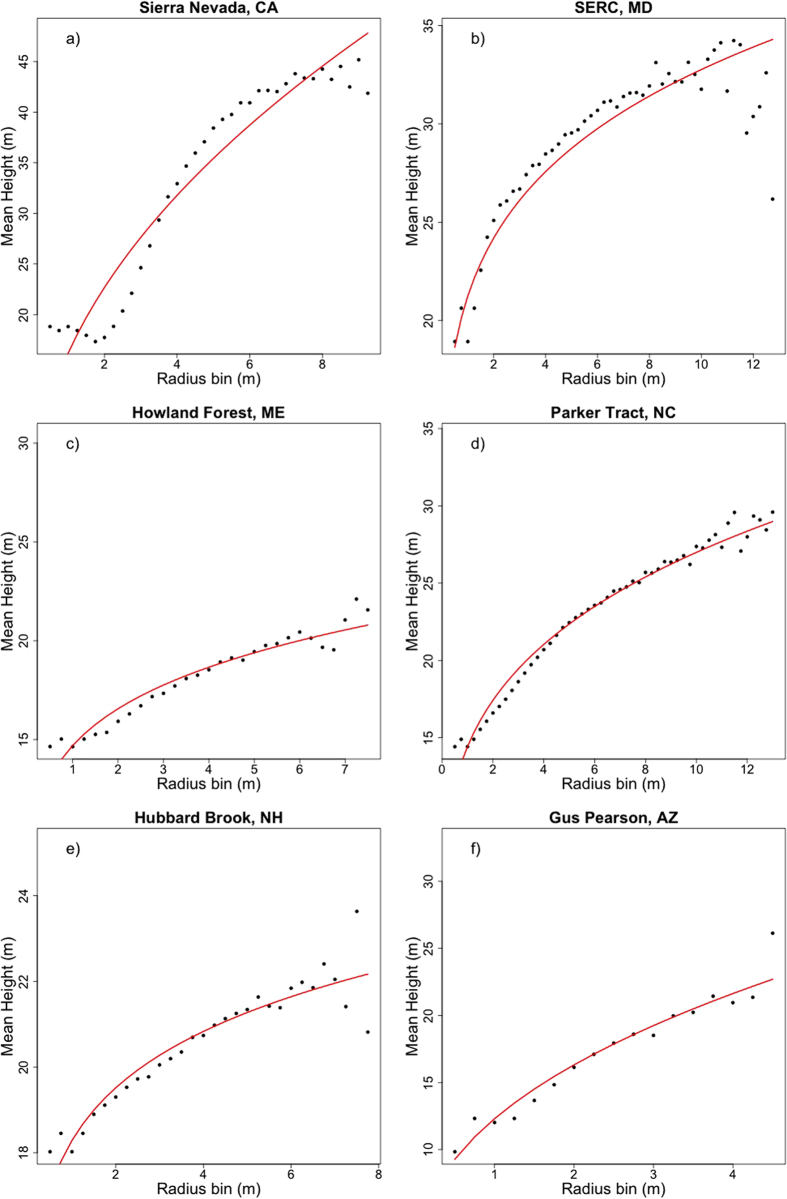
The population-level allometric equations using all delineated crowns. The black dots represent the median tree height in each 25 cm crown radius bin, roughly representative of the black bars in Fig. 1. The lines are power law curves fit to each distribution. The parameters of these curves are assumed to represent the true, or site-level allometry at each site.

In each of our six study sites, the relationship between crown radius and tree height is highly variable (Fig. 1). We simplify these relationships as power laws describing the median height in a 25 cm crown-radius bin. These site-level equations (Fig. 2) are developed with the full sample size at each site. We then extract samples of trees from our LiDAR datasets, and fit power laws relating median height to crown radius bin for various sample sizes, in an attempt to assess the sensitivity of allometric parameters to sample size. We use two different sampling strategies for this analysis: random sampling and stratified sampling (see Methods). We assess the sensitivity of the two power law parameters, the exponent, α, and the scalar, β, for each sampling strategy.

#### Random Sampling

**Figure 3:**
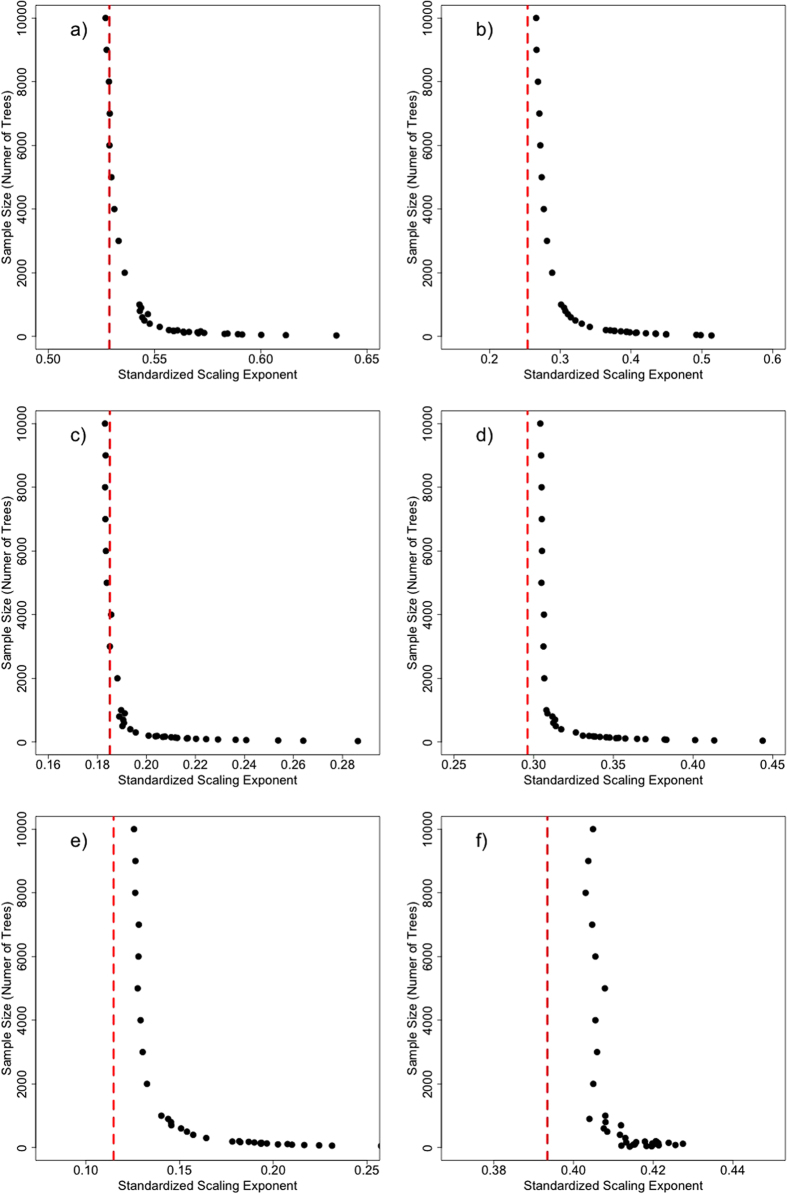
The average allometric power law exponent, α, for a given sample size from the random sampling approach at (**a**) Teakettle, (**b**) SERC, (**c**) Howland, (**d**) Parker Tract, (**e**) Hubbard Brook, and (**f**) Gus Pearson. In general, the exponent decreases as the sample size increases, approaching an asymptote representing the true or site-level allometry.

**Figure 4:**
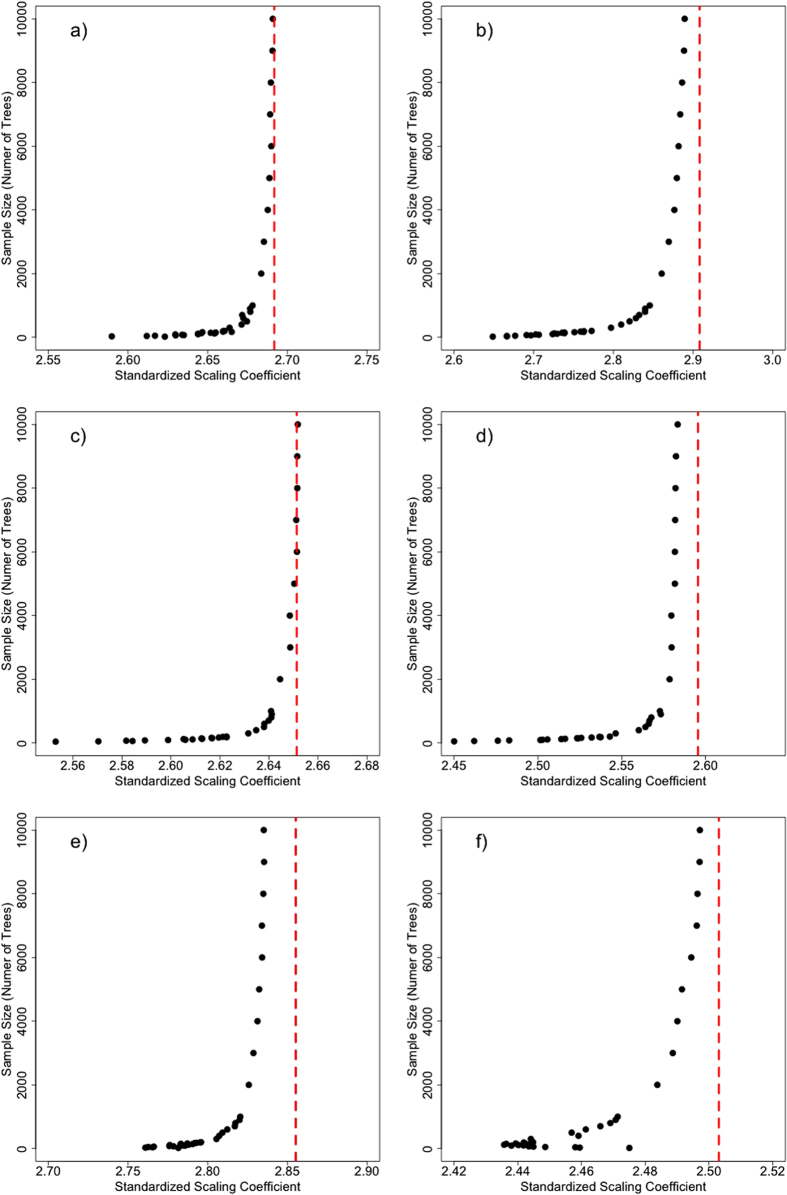
The average allometric power law scalar, β, for a given sample size at (**a**) Teakettle, (**b**) SERC, (**c**) Howland, (**d**) Parker Tract, (**e**) Hubbard Brook and (**f**) Gus Pearson, using the random sampling approach. In general, the scalar increases as the sample size increases, approaching an asymptote representing the true or site-level allometry.

**Figure 5:**
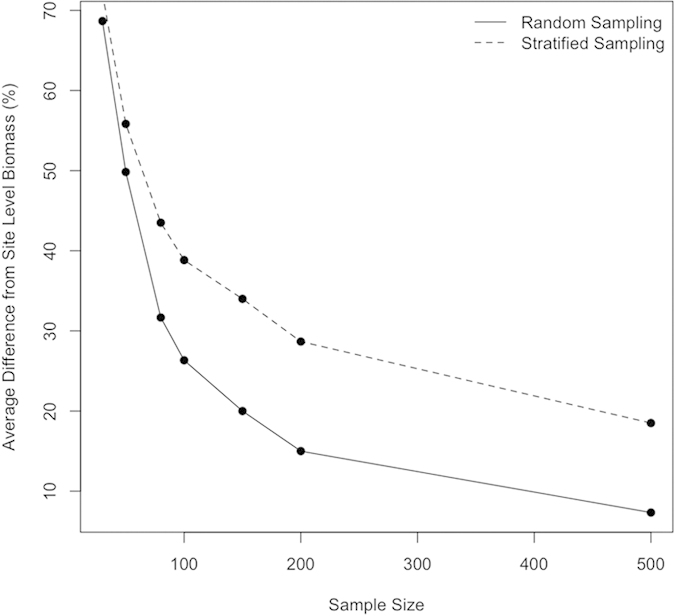
The deviation from the site-level biomass estimated using the full sample size, as a function of sample size. Random sampling yields lower overestimations, on average, and approaches zero bias with a sample size of 500, while 500 samples retains an ~20% overestimate in site-level biomass for the stratified sampling approach.

As sample size increases, there is a consistent decrease in α (Fig. 3), and a corresponding increase of β (Fig. 4), with both values approaching an asymptote at the population value (represented by the vertical red lines on Figs. 4 and 5). These trends are consistent across study sites. Taken alone, an overestimation of α would yield an overestimation in height for large crown radii, while an underestimation of β would yield an underestimation of height.

#### Stratified Sampling

For our stratified sampling approach, our results are generally similar to those found with random sampling, suggesting that the trend of overestimating α, and underestimating β at small sample sizes is not a function of sampling strategy. However, the fitted parameters converge to different values using stratified sampling than random sampling. In most sites, parameters approach higher values of α and lower values of β. This represents a more linear relationship (higher α) between height and crown radius, with a shallower slope (lower β). Deviations from site-level parameters are generally larger with stratified sampling than with random sampling.

### Carbon Implications

To address expected biomass implications, we use equation (7) (see Methods) to estimate tree biomass as a function of crown radius and our allometric parameters, α and β. Summing these tree level estimates over the number of trees found in each study area, we estimate the deviation from site-level biomass as a function of sample size for random sampling (Table 1) and stratified sampling (Table 2). From here forward, we refer to error as this observed deviation from site-level estimated biomass. Results vary considerably across our six study sites and between our two sampling strategies. However, we generally overestimate site-level biomass when using allometric equations developed from small sample sizes. The mean site-level overestimations are presented as a function of sample size in Fig. 5.

Our analysis shows that the parameterization of allometric equations varies considerably as a function of sample size. Our results corroborate the findings of Chave *et al.* (2004) and Hunter *et al.* (2015) but suggest that in some forests, the potential impact of using allometric models based on small sample sizes for biomass prediction extends well above Chave’s 30% error, in some cases causing overestimations of more than double the presumed biomass. This overestimate of biomass is caused by the under sampling of large trees, because of the non-linear relationships between both crown radius and height, and crown radius and biomass. When we only sample smaller trees, we fit more linear relationships. When extended over the full tree size distribution of an area, this overestimates the height and biomass of large individuals. It is therefore important to sample the full tree size distribution over which allometric equations will be applied.

There are three important trends visible in Tables 1 and 2. First, as sample size increases, errors in biomass estimation decrease because a higher number of large trees are sampled (Fig. 5). Importantly, in all sites but Gus Pearson, biomass is overestimated with small sample sizes, and this overestimation decreases as more trees are sampled. Second, stratified sampling typically yields lower overestimations than random sampling for the smallest sample size (n = 30), because it samples the size distribution in each pseudo plot, ensuring some small and some large trees are included in each sample. However, stratified sampling yields comparable or higher overestimations for larger sample sizes (n > 80). Third, there is considerable variability in overestimations between sites, and this variability also decreases with increasing sample size.

In general, random sampling is more accurate than stratified sampling for larger sample sizes (n > 80). This is because the stratified sampling skews the sampling towards the largest and smallest trees (tails of the distribution) allowing a more representative sample for the smallest sample sizes, but over sampling large and small trees as the sample size increases.

Focusing on the results from stratified sampling, as these are likely more representative of real world forest mensuration, we see that at a sample size of 30, consistent with the average sampling from Jenkins *et al.*, (2003), there is an overestimation of site level biomass ranging from 20% at Hubbard Brook to 193% at Teakettle. The two largest overestimations, at SERC and Teakettle, are likely because these are the sites with the largest trees, and biomass overestimation will increase with tree size. Therefore although the fitted parameters deviate more from site level values at Hubbard Brook than at Teakettle, the higher proportion of large trees at Teakettle yields a larger site level overestimation of biomass.

## Discussion

### Improving Allometric Equations

We show that allometric parameters are sensitive to sample size, and that parameters are systematically biased as a function of small sample sizes across six forested sites in the United States. Our analysis on the carbon implications of these results suggests that we may be systematically overestimating field carbon stocks in North America through the application of allometric equations developed with small samples sizes. This problem has been difficult to address in the past because of a lack of destructively sampled trees^34^, and consequently we have not been able to quantify the potential carbon implications of small sample sizes in temperate systems. Nonetheless, the magnitude of these biases confirms for temperate forests what others have suggested for tropical forests: that a much more thorough analysis of forest allometry is needed. These results may also have implications for allometric equations developed to estimate below-ground biomass, which are also based on small sample sizes of destructively sampled root systems^35^.

In this study we demonstrate the utility of LiDAR data for population-level analyses of forest structure. We rely on data acquired in North America where there is wide availability of high point density LiDAR datasets and relatively simple forest structures that allow the extraction of individual crown information from the LiDAR data. This research would be more difficult to conduct in tropical forests, where complex, intertwined canopies are more problematic to delineate. However, improvements in delineation algorithms, wider availability in high point density LiDAR datasets, and fusion with terrestrial scanning LiDAR could soon enable a complimentary study focused specifically on tropical systems.

We conclude that past sample sizes have been insufficiently large to accurately parameterize allometric relationships in temperate forests. The same technology we use to illuminate the problem of small sample sizes could also be used to remedy it. The limiting factor here has always been the destructive sampling of trees, and we believe that destructive sampling may no longer be a requirement, given recent advances in LiDAR technologies, particularly highly portable ground-based LiDAR^36^. Highly precise estimates of individual tree volumes are increasingly available^37^. These estimates do not require the destructive sampling of trees, and can be conducted in a systematic fashion in the field. As such, much higher sample sizes can be acquired, including samples of very large trees for which destructive sampling would be logistically impractical. In tandem with an increased understanding of the variability of wood densities^38^,^39^, these individual tree volume measurements could be used to produce the sample sizes necessary to reduce biomass bias at the individual tree level. With appropriate sampling and campaign design, a system could be developed to sample *in situ* tree volume across environmental gradients, providing a potential solution to outstanding problems related to forest allometry.

## Methods

### Study Areas

We use forested areas in the United States, selecting sites with a range of species compositions, ages, and management practices in order to determine how variable the effects of sample size are on allometric equations across disparate conditions. High-resolution airborne LiDAR data were acquired over each study site and processed through an individual tree detection algorithm^27^.

#### Teakettle Experimental Forest, Sierra Nevada, California

Teakettle is located within Sierra National Forest in the Sierra Nevada Mountain range in California. Dominant species include *Abies concolor (*white fir), *Pinus ponderos* (ponderosa pine), *Abies magnifica* (red fir) and *Quercus kelloggii* (California black oak). The elevation range of the site is approximately 1000 m to 2500 m above sea level, with aboveground biomass values averaging 200 Mg ha^−1^ with individual tree values up to 20 Mg tree^−1^. The forest is mature, with rocky outcrops intermixed between clusters of trees. Fire is the primary disturbance affecting the ecosystem.

#### SERC, Maryland

The Smithsonian Environmental Research Center (SERC) study site is located near Edgewater, Maryland, adjacent to a sub-estuary of the Chesapeake Bay. The area is generally comprised of two forest types: mature secondary upland forest, and lowland forests. Dominant species in the upland forest include *Liriodendron tulipifera* (tulip poplar), *Fagus* (beech), several species of oak, and hickory, with mid canopy *Acer rubrum* (red maple) and *Nyssa sylvatica* (black tupelo) and understory *Carpinus caroliniana* (American hornbeam), *Lindera benzoin* (spicebush) and *Asimina triloba* (paw-paw). Dominant species in the lowland areas are *Fraxinus* (ash), *Platanus occidentalis* (sycamore), and *Ulmus americana* (American elm). Both the upland and the floodplain forests have been relatively undisturbed for approximately 120 years.

#### Parker Tract, North Carolina

The Parker Tract study site is located near Plymouth in North Carolina. It is largely a commercially managed loblolly pine plantation (*Pinus taeda)* although some stands have a mixed composition, containing native broadleaf species. One segment of the site is retained as natural forest.

#### Gus Pearson Natural Area, Arizona

Gus Pearson is located within the Fort Valley Experimental Forest in Arizona. The site is comprised primarily of ponderosa pine (*Pinus ponderosa*). The primary disturbance at this site is from thinning and burning experiments that have effectively decreased the frequency of small trees, shifting the tree size distribution toward larger individuals^40^.

#### Howland Research Forest, Maine

The Howland Research Forest is a conifer-dominated mixed forest located in central Maine. The site is dominated by Red Spruce, Eastern Hemlock, and White Cedar. The site is mature, with stand ages ranging from 45 to 130 years. Although it has been used for studying the effects of acid rain and carbon flux, management has not significantly altered the natural tree size distribution.

#### Hubbard Brook Experimental Forest, New Hampshire

Hubbard Brook is the largest study area we examined. The area is a mixed forest site located near Woodstock, New Hampshire, and is primarily dominated by second-growth northern hardwoods, red spruce, and balsam fir. The site exhibits considerably ecological variation across topographic gradients^41^.

### LiDAR Data

LiDAR data at four sites were acquired by NASA Goddard’s LiDAR, Hyperspectral and Thermal Imager (G-LiHT^42^). G-LiHT uses a 300 kHz multi-stop scanning-LiDAR operating at 1550 nm with a 60° field of view and 10 cm diameter footprint. At Teakettle, LiDAR were collected by the University of Florida with an Optech Gemini ALSM unit, operating at 1064 nm with a 100–125 kHz frequency, a 25° scan angle, and 50–75% overlapping swaths. At Hubbard Brook LiDAR were collected by the Canaan Valley Institute, flying an Optech instrument operating at 1064 nm with a 18° scan angle, 100 kHz frequency, and 15 cm footprint. The sites were generally flown during the snow-free, leaf-on season. The collection dates were in the spring of 2013 at Teakettle, June of 2012 at SERC, July of 2011 at Parker Tract, March of 2013 at Gus Pearson, June of 2012 at Howland Forest, and fall of 2009 at Hubbard Brook. Sites were typically flown from an altitude of 335 m with 50% overlap in north-south and east-west directions to achieve a mean return density of up to 50 laser pulses m^−2^.

### Canopy Delineation

Individual tree metrics are derived from the LiDAR point cloud through a multilayered canopy delineation algorithm^27^ that is capable of accurately extracting crown dimensions from both coniferous and deciduous trees, and from understory and overlapping crowns. In a previous study, we tested this algorithm in two of the six study areas used in this analysis, and found that it performed best in open conifer forests, but even in closed-canopy deciduous forests was able to accurately extract ~70% of dominant crowns^27^. Although errors of omission and commission will always occur when attempting to detect every tree across a landscape, we have demonstrated that the tree size distributions gleaned from the LiDAR delineation match those found in the field datasets (Supplementary Figures 1 and 2). Because the algorithm has been tested in both conifer and deciduous high-biomass forests and can be run without requiring local parameterization, it is ideal for our study as it allows a comparison of tree crowns and heights across a variety of forested ecosystems. The algorithm is run on the Pleiades supercomputer at NASA Ames as part of the NASA Earth Exchange.

### Allometric Equation Fitting

Individual tree heights and crown radii are extracted from the LiDAR point cloud at each study area, all of which include a very high number of delineated crowns with differing tree size distributions. To remove the influence of tree size distribution or outliers on our analysis, we bin our data by calculating the median tree height in 0.25 m crown radius bins. For a more thorough discussion of the effects of binning, refer to the Supplementary Information (SI Figs. 3 and 4). Log-log linear models provide the best descriptions of the relationship between stem diameter and Height^32^. Accordingly, we fit a model in the form of a power law using the full tree dataset at each study site to produce a set of site-level scaling parameters. Each power law model is fit using Model 2 regression on log transformed, binned data with ranged major axis (RMA). RMA is used because errors exist in the estimation of both tree heights and radii^43^. The relationship between height and crown radius is given by:


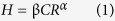


where *H* is height, *CR* is crown radius, and β and α are the scaling parameter and fitted exponent, respectively. The allometric parameters that are calculated using the full population of delineated trees at each site are assumed to be the true scaling parameters representing the allometry at each site. We extract samples from the full dataset to assess the influence of sample size on the fitted parameters. From the literature, studies either do not report how they selected trees to fell, or report that they selected trees that appeared representative of the apparent size distribution. To represent both random selections of trees and stratified selections of trees, we use two sampling strategies in this paper: (1) random sampling, and (2) stratified random sampling.

#### Random sampling

We iteratively generate samples from our full dataset in each study area, selecting trees randomly with sample sizes increasing by 5, from n = 10 until the full number of trees at each study site. For each randomly sampled set of trees, we follow the model fitting procedure used for the site-level analysis, as outlined above. As random sampling produces highly variable fitted parameters, we iterate the random sampling 500 times for each sample size, and calculate the average parameter over the 500 iterations to produce a single average estimate of α (the scaling exponent) and β (the scaling coefficient) for each sample size.

#### Stratified Random Sampling

In an attempt to simulate a more realistic approach to sampling in the field, we also apply a technique that samples trees that are spatially clustered. We adopt a stratified sampling scheme that is approximately representative of field mensuration. It should be noted, however, that sampling for biomass equation development varies considerably, often based on arbitrary decisions made in the field. In our stratified sampling approach, we simulate sampling at a pseudo plot-level. We randomly select locations within each study area, and extract all trees in a 30 m plot corresponding to each randomly selected location. We then select five trees from within each plot, taken at the 10^th^, 30^th^, 50^th^, 70^th^, and 90^th^ percentiles of crown radius. Sample size is increased by randomly selecting more plot locations, and extracting five trees from each new plot. For each sample size, the data are pooled, binned, and a model is fit following the methods for the site-level and random sampling analysis.

### Carbon Implications of Small Sample Sizes

As discussed, we explicitly test the allometry between crown radius and height rather than between stem diameter and biomass. We use crown radius as a proxy for stem diameter, with the assumption that a) there is a linear relationship between diameter and crown radius, and b) that we are correctly extracting crown radii. These assumptions are satisfied with an analysis of the data presented in Supplementary Figures 1 and 2, which show similar size distributions from field collections of stem diameters and corresponding LiDAR collections of crown radii. In an attempt to translate our results to the relationship between stem diameter, D, to biomass, M, we use regional empirically fitted exponents from the literature (for D to M) or the Forest Inventory Analysis (FIA) dataset (for D to height):


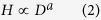



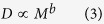



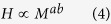



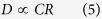


where *H* is height, *D* is stem diameter at breast height, *M* is aboveground tree biomass, and *CR* is crown radius. Combining these three equations allows us to translate biases in the estimation in exponents relating *CR* and *H* to the relationship between *CR* and *M*.


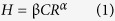



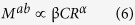



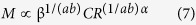


where *a* and *b* are the regional empirically fitted exponents in equation (2) and equation (3), respectively, and alpha and beta are the parameters we fit in this study, which vary with sample size. To estimate site-level biomass, we use equation (7) to calculate the individual tree level biomass for each 25 cm *CR* bin in each study site, and multiply by the number of trees within that bin. The biomass in all *CR* bins is summed to estimate site level biomass. First, we estimate site-level biomass with the site-level allometric parameters, followed by parameters corresponding to sample sizes of 30, 50, 80, 100, 150, 200, and 500 for each sampling strategy (random and stratified). The biomass estimates corresponding to each sample size are divided by the biomass estimate using the site-level allometry to give a percentage over- or underestimate of biomass for each site as a function of sample size. Note that the effects of binning will be the same on the population-level and the sampled analyses, and therefore will not affect our results.

#### Regional Allometric Parameters

In order to translate between our observed allometry, equation (1), and potential biomass implications, equation (7), we rely on the use of empirically derived regional allometries relating crown radius to stem diameter, equation (5), stem diameter to height, equation (2) and stem diameter to biomass, equation (3). We assume that crown radius scales linearly with stem diameter^31^,^44^, and rely on the assumption that regional allometries are applicable to our study sites. We take exponents for equation (2) from the freely available U.S. Forest Service’s Forest Inventory Analysis (FIA) dataset by extracting individual stem diameter and height measurements for the county corresponding to each study location and fitting an empirical allometric equation in the form of a log-log linear relationship, assuming that the slope represents the exponent in equation (2) These exponents are presented in Table 3.

For equation (3), stem diameter to biomass, we rely on recent generalized allometric equations applicable to U.S. forests^18^. These generalized equations are based on a meta analysis, combining species-specific localized equations into more generally applicable ones, based on wood specific density and species structural form. We select the generalized allometric equation or equations in each study site corresponding to the dominant species available. Table 4 provides information pertaining to the selected species at each site.

For both sets of empirically fitted allometries there is a scalar and an exponent, however we only use the exponent in this analysis. This is because we do not focus on the precise estimation of biomass at a given study site, but on the over- or underestimation of biomass as a function of sample size. Therefore the scalars for each of the equations (2, 3, 4, 5, 6), 8 combine to a single scalar in equation (7) that does not impact the over- or underestimations reported in Tables 1 and 2.

## Additional Information

**How to cite this article**: Duncanson, L. *et al.* Small Sample Sizes Yield Biased Allometric Equations in Temperate Forests. *Sci. Rep.* **5**, 17153; doi: 10.1038/srep17153 (2015).

## Supplementary Material

Supplementary Figures

## Figures and Tables

**Table 1:** Percentage deviation from site-level biomass estimation as a function of sample size, using random sampling.

Sample n	Teakettle	SERC	Howland	Parker	Hubbard Brook	Gus Pearson
30	48	193	32	109	34	−4
50	32	165	20	64	23	−5
80	25	103	11	42	13	−4
100	20	93	9	32	10	−6
150	18	72	6	22	7	−5
200	16	55	3	15	6	−5
500	12	34	0	5	−1	−6

Values are presented as % under or overestimation. The largest deviations at small sample sizes are at the SERC, Parker Tract and Teakettle Sites.

**Table 2:** Percentage deviation from site-level biomass estimation as a function of sample size, using stratified sampling.

Sample n	Teakettle	SERC	Howland	Parker	Hubbard Brook	Gus Pearson
30	143	147	40	78	16	9
50	106	122	24	67	10	6
80	92	86	20	51	8	4
100	93	68	17	36	13	6
150	88	57	13	34	7	5
200	78	47	12	31	5	−1
500	65	29	6	15	1	−5

Values are presented as % under or overestimation. The overestimations in all but Teakettle are lower than with random sampling with a sample size of 30, but higher as sample size increases.

**Table 3:** Empirically-derived regional coefficients relating stem diameter to height from the Forest Service’s forest inventory dataset, fit to all individual tree data for the county corresponding to each field site.

Site	State	County	Scalar	Exponent	R^2^
Teakettle	California	Fresno	−0.07	0.785	0.80
Parker Tract	North Carolina	Washington	1.01	0.586	0.85
Hubbard Brook	New Hampshire	Grafton	0.73	0.636	0.49
Howland Forest	Maine	Penobscot	0.87	0.579	0.55
Gus Pearson	Arizona	Coconino	−0.53	0.855	0.50
SERC	Maryland	Anne Arundel	0.88	0.643	0.66

The Exponents in this table are used as the *a* coefficients in equation (2).

**Table 4:** Average allometric parameters relating stem diameter to biomass for the dominant tree species found at each study site.

Site	Group	Taxa	Scalars	Exponents	Average Exponent
Teakettle	Conifer	Abies < 0.35 spg Pinus > 0.45 spg	−2.3123, −2.6177	0.4259, 0.4060	0.4156
Parker Tract	Conifer	Pinus > 0.45 spg	−3.0506	0.3779	0.3779
Hubbard Brook	Conifer/Hardwood	Abies < 0.35 Picea > 0.35 Fagae, deciduous Betulaceae, 0.4–0.49	−2.3123, −2.1364, −2.0705, −2.2271	0.4259, 0.4304, 0.4097, 0.4079	0.4182
Howland Forest	Conifer	Picea > 0.35 spg Cupressaceae < 0.3 spg Tsuga < 0.40 spg	−2.1364, −1.9615, −2.3480	0.4304, 0.4748, 0.4188	0.4401
Gus Pearson	Conifer	Pinus < 0.45 spg	−2.6177	0.4059	0.4059
SERC	Hardwood	Fagae, deciduous	−2.0705	0.4097	0.4097

The average exponents are used as the *b* coefficients in equation (3). Spg is the specific gravity of wood for a given taxa.
