# Supplementary material for: Small Sample Sizes Yield Biased Allometric Equations in Temperate Forests
Source: Sci Rep. 2015 Nov 24;5:17153. doi: 10.1038/srep17153 (PMC4657147; doi:10.1038/srep17153)
Supplement: Supplementary Figures [file srep17153-s1.doc]

Supplementary Information for:

Small Sample Sizes Yield Biased Allometric Equations in Temperate Forests

Authors: Duncanson, L.,*^1,2^ Rourke, O.^3^, & Dubayah, R.^1^

1. Department of Geographical Sciences, University of Maryland, College Park, 2181 Lefrak Hall, University of Maryland, College Park, MD, 20742
2. Biosciences Lab, Code 618, NASA’s Goddard Space Flight Center, Greenbelt, MD 20771.
3. AMSC, Department of Mathematics, University of Maryland, College Park, 0209 Mathematics Building, University of Maryland, College Park, MD, 20742

Corresponding author: Laura Duncanson, 2181 Lefrak Hall, University of Maryland, College Park, MD, 20742. Telephone: 1-301-256-4302. Email: [lduncans@umd.edu](mailto:lduncans@umd.edu).

**LiDAR Delineation Performance**

Our Lidar crown delineation was tested with field data in two of the six study areas used in this analysis. Supplementary Figure 1 shows a comparison between the extracted tree crown distributions from Teakettle and SERC (a and c) and the corresponding stem diameter distributions from the field datasets (b and d). In combination with the quantile-quantile plots provided in Supplementary Figure 2 we demonstrate that our delineation algorithm adequately captures a suitable proxy for stem diameter distribution in two of our most disparate study sites. For further details on the accuracy assessment of our crown delineation algorithm please refer to Duncanson et al., 2014.


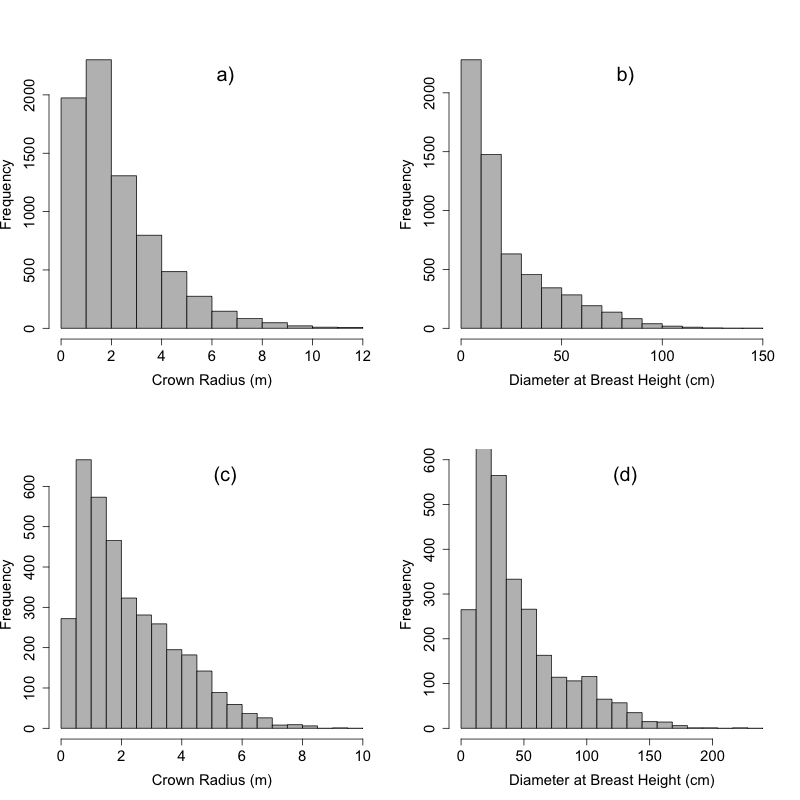


Supplementary Figure 1. The extracted distributions of crown radii (a, c) generally match the distributions of stem diameters (b, d) in field plots at SERC and Teakettle, respectively. This demonstrates that conclusions drawn regarding the sampling of crown radius are theoretically transferable to sampling of stem diameter.
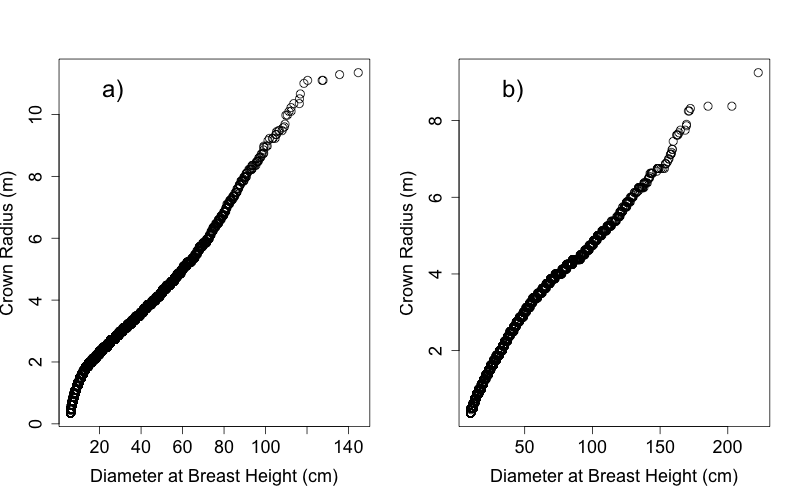


Supplementary Figure 2. Quantile-quantile plots allow a comparison of distributions of crown radius to stem diameter at SERC (a) and Teakettle (b). The near linear relationships here demonstrate that crown radius and stem diameter distributions are comparable, although slight deviations are seen in very small and very large trees.

**The Importance of Binning Data**

For our fitting of allometric relationships between crown radius and height we performed a binning of the data, in which we fit relationships to the median height value in 25 cm crown radius bins, as visualized in Fig 2. We decided to bin the data rather than fit allometric equations to the raw data points for two reasons. First, to remove the influence of outliers on the allometric parameterization, as power law fits are known to be sensitive to outliers and our delineation algorithm is imperfect. Second, and more importantly, to adjust for the tree size distribution found in these forests. If we fit models to the raw data, the models would be more accurate for the more prevalent small stems and less accurate for the rare, larger stems. This phenomenon is demonstrated in Supplementary Figure 3, where we show the difference between fitting models to the raw data (red lines) and binned data (blue lines). The blue lines better capture the relationships between crown radii and height in the larger size classes, and therefore we elected to bin our data throughout this analysis.

In order to demonstrate that the binning procedure itself does not cause the relationship between sample size and allometric parameters, as seen in Figs 3 and 4, we repeated the analysis on the raw data and found similar patterns (Supplementary Figure 4). Therefore we conclude that the overestimation of power law exponents with small sample sizes is not a result of our binning procedure but a result of sampling from a power law type distribution where it requires large sample sizes to adequately sample larger stems.


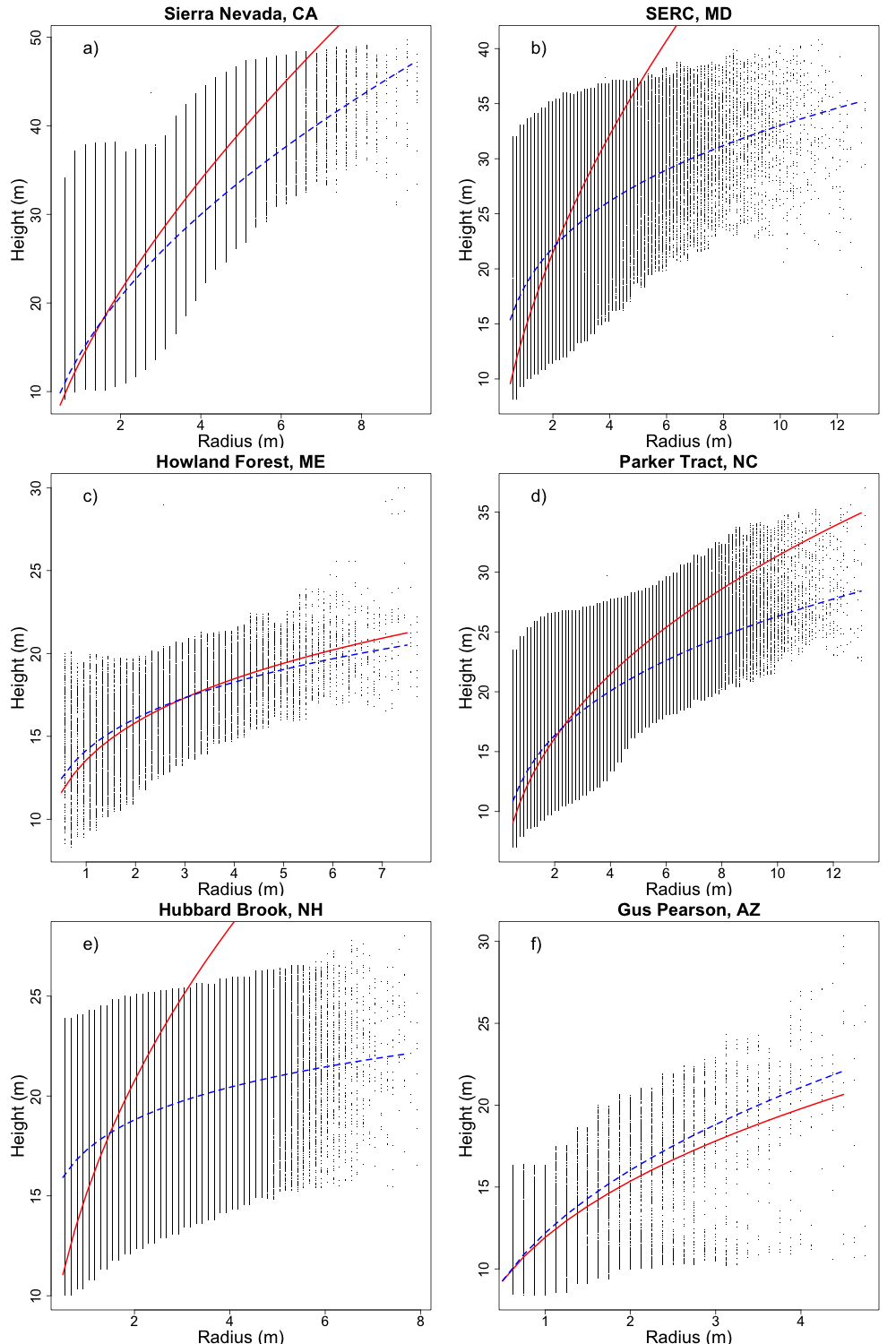


Supplementary Figure 3. The relationships between crown radius and height when fit to the raw data (red line) or binned data (blue line). We show here that binning the data is important to capture the relationships between crown radius and height for larger trees.


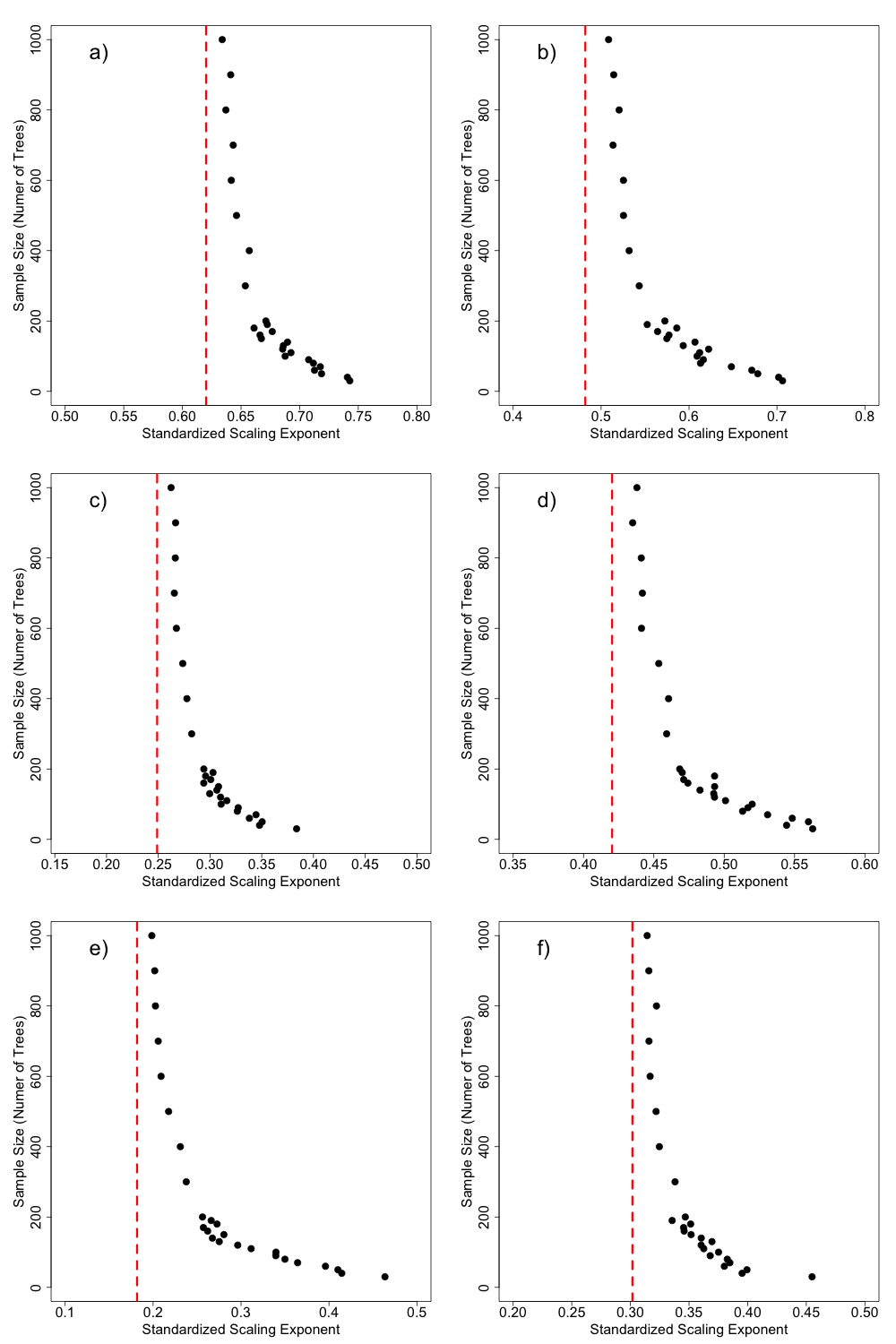


Supplementary Figure 4. The relationship between fitted exponents (alpha) to sample size using random sampling and raw, unbinned data. The shape of the relationship is akin to that shown in Fig 3, but exponents constrain to a different value at high sample sizes because they are constraining to a different relationship (the red line rather than the blue line in Supplementary Figure 3).

**
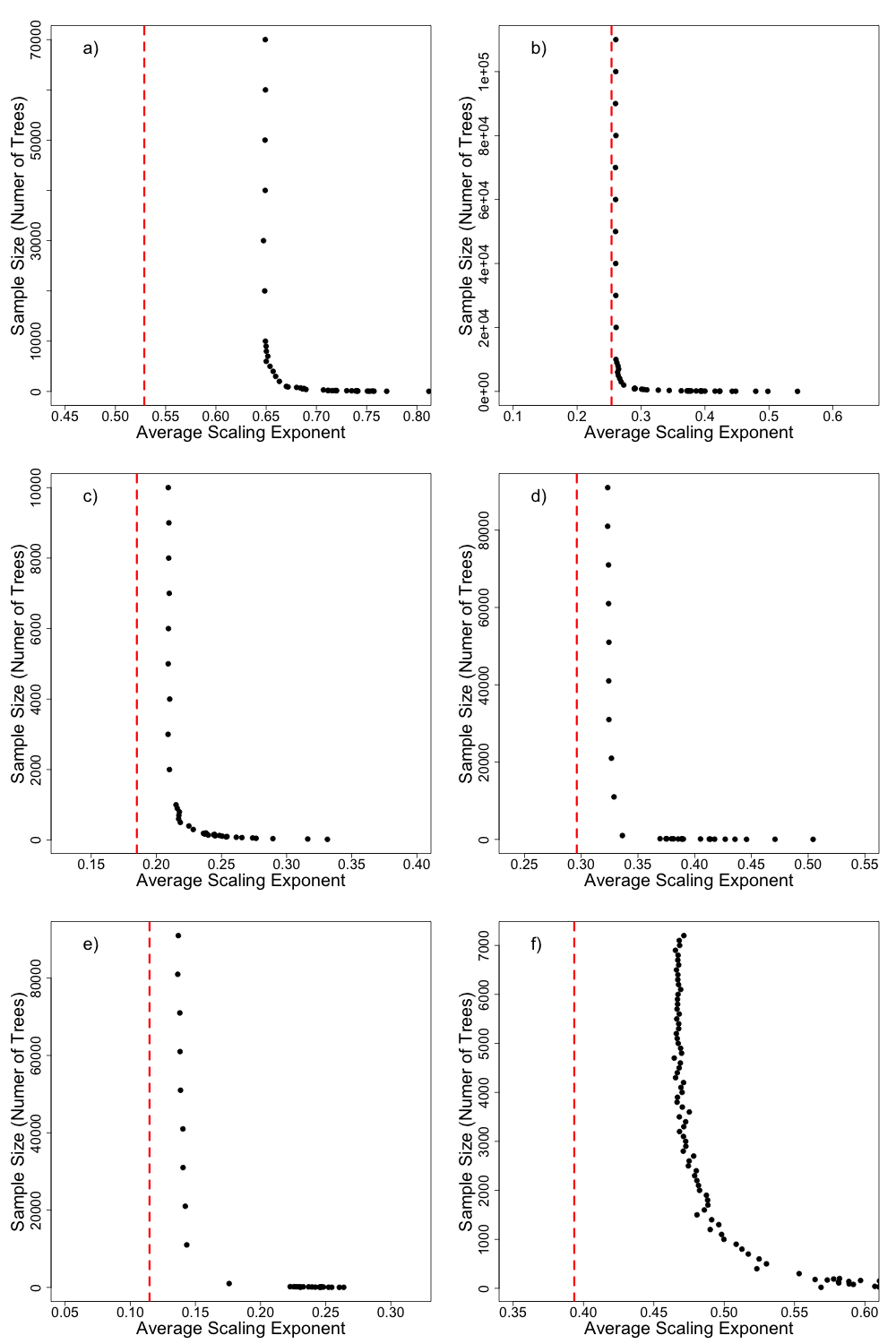
**

Supplementary Figure 5. Stratified sampling yields allometric exponents that converge to generally higher values of alpha as sample size increases. The dotted lines here represent the population-level value of alpha, the scaling exponent at a) Teakettle, b) SERC, c) Howland, d) Parker Tract, e) Hubbard Brook, and f) Gus Pearson.

**
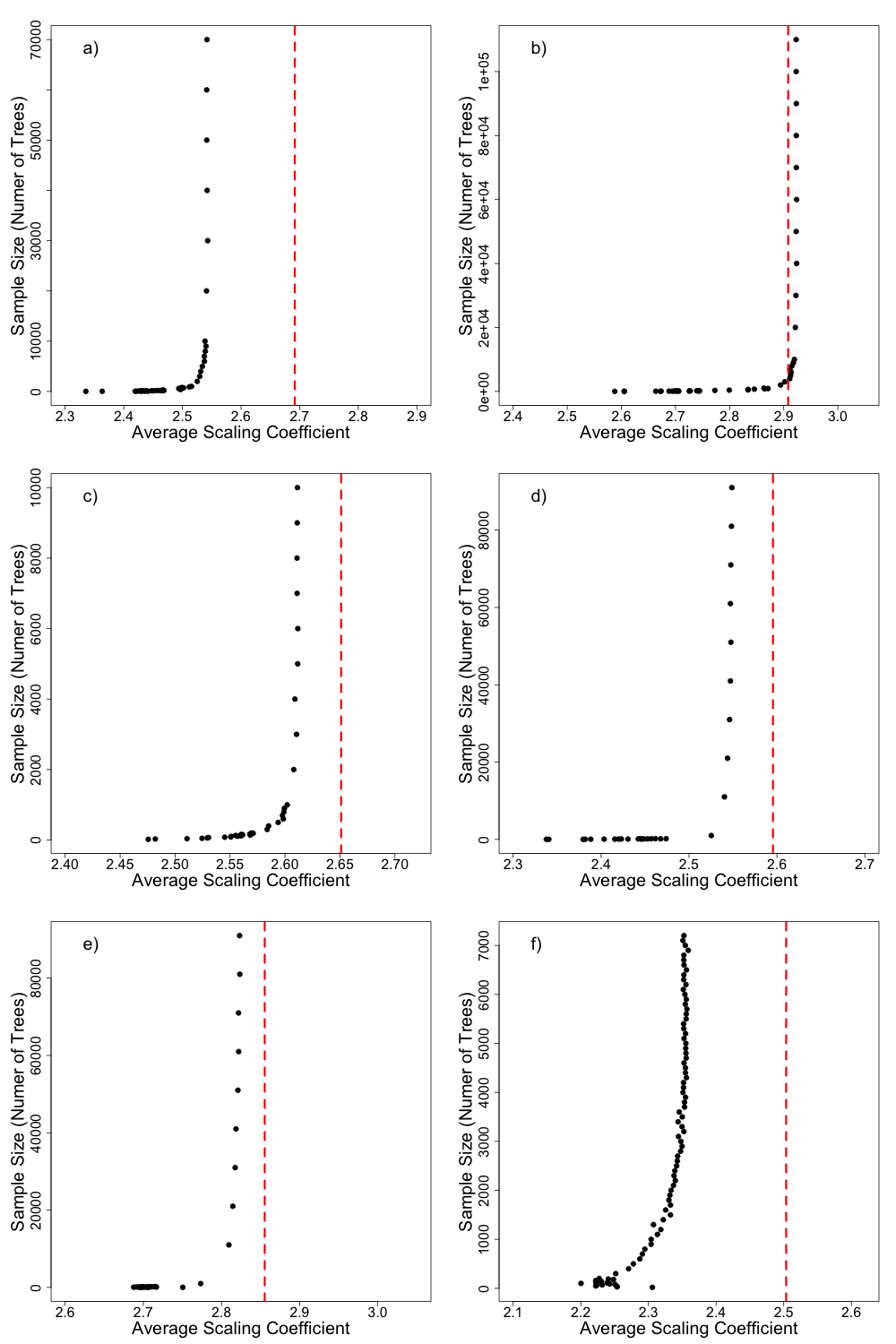
**

Supplementary Figure 6. Stratified sampling yields allometric scalars that converge to generally lower values of beta as sample size increases. The dotted lines here represent the population-level value of beta at a) Teakettle, b) SERC, c) Howland, d) Parker Tract, e) Hubbard Brook, and f) Gus Pearson.
